# Support of students by academics in a nursing foundation programme at a university in the Western Cape

**DOI:** 10.4102/curationis.v41i1.1927

**Published:** 2018-08-07

**Authors:** Annelize D. Daniels, Karien Jooste

**Affiliations:** 1 Department of Nursing, Cape Peninsula University of Technology, South Africa

## Abstract

**Background:**

Owing to the inadequate schooling system and the under-preparedness of students in South African high schools, Higher Education Institutions are faced with students who do not meet the minimum criteria for acceptance into a mainstream programme and need support from an extended foundation programme.

**Objectives:**

The study described the support of students by lecturers in an extended 5-year nursing foundation programme.

**Method:**

A qualitative, exploratory and descriptive design was applied. Purposive sampling was conducted, and eight nursing students who completed the foundation year took part in semi-structured individual interviews. Each interview took around 30 min and was digitally recorded. Data were transcribed verbatim and analysed using open coding. Ethical principles and trustworthiness were maintained throughout the study.

**Results:**

The findings of this study indicated that experiences varied on a foundation programme. Participants expressed support in a nurturing environment in which the lecturers were open and approachable with a positive attitude. Lecturers could support students by being caring and advising in learning methods around the programme. Lecturers could focus on the method of peer support which should be encouraged beyond the programme so as to provide a sense of camaraderie amongst students.

**Conclusion:**

Data revealed that support of lecturers in a foundation programme needs creative methods to make the learning environment nurturing for students. This provides for students from diverse backgrounds the opportunity to prepare for their studies at a university level. Insights gained from this study, which highlight the importance of supporting foundation students, could benefit all nurse educators offering foundation programmes.

## Introduction

A Department of Nursing in a Faculty of Health Sciences at a university in the Western Cape had requests from matriculates not meeting the required university grade to enter the nursing 4-year degree programme. The university has a focus on disadvantaged students and was aware of the post-apartheid South Africa’s education system that has perpetuated the racial and class inequalities of the previous regime. The majority of working class black students come from poorly resourced schools and are ill-equipped to make the university grade (Hutchings 2017). To address this issue, the Department of Education (DoE) has previously developed policies that encourage transformation within the new educational system (The Education White Paper 3: A Program for the Transformation of Higher Education, DoE 1997). The basic programme was offered over 4 years, and in 2005, it was decided in the Faculty of Health to offer an extended 5-year programme for nursing, for students to complete the first year over 2 years. These students could then enter with a standard point score (Admission Point Score of 26 instead of 28 with similar subjects as required for the 4-year programme).

In primary and secondary education, South African students underperform even relative to the BRICS (Brazil, Russia, India, China and South Africa, the five major emerging national economies) peer countries such as East Asia and Russia. Students, because of a dysfunctional educational system, often arrive at university with massive academic deficits. Although this dilemma is not easy to address, bridging programmes such as a foundation programme can provide an answer (Govender 2013).

According to research, from the late 1950s to date, the white and Asian populations have seen a proportional rise in the number of students who successfully passed matric, entered a tertiary education system (university) and completed their bachelor’s degrees (Destiny Man Reporter 2017). However, not enough black and Coloured^1^ children are successfully completing their higher education, compared to their white and Asian counterparts (Destiny Man Reporter 2017). The University of Cape Town therefore implemented a new admissions policy model, addressing the incorporation of race as one of several factors to consider in assessing an applicant’s historic disadvantage (Newsroom 2014). This came along with universities starting to look at foundation programmes to support disadvantaged students.

Seeth (2017) indicates that there has been an increase in enrolments of black students at tertiary institutions over the last few years. Although the universities have increased their intake of students of colour, the universities are poorly prepared to deal with the large intake of students (Seeth 2017). Although this is happening, support to students becomes more and more important. Therefore, one key to overcome these factors, which contributes to inhibiting student learning, is not only a matter of increasing access (CHE 2013:9).

The challenge of foundation programmes is to provide a foundation pathway between matric and university entry requirements for undergraduate nursing studies (Monash South Africa 2018). The aim of these programmes is to support students to allow an extra year for bridging the ‘educational gap’ (Kloot, Case & Marshall 2008:799).

A Bachelor Nursing Foundation Programme (hereafter called foundation programme) has been offered by a Department of Nursing at a university in the Western Cape since 2007, in order to meet the needs of under-prepared students for the 4-year degree programme, as well as to address transformation of the programme. Students who applied to the university were mainly from under-served communities, and all students who are a first entrance student at a higher education institution may not be prepared for the style of instruction in a higher education institution. The Department of Nursing therefore started to offer a foundation nursing programme with an intake of 50 students per year with the ratio of lecturers to students being 1:25. The lecturers also partook in clinical supervision, and clinical supervisors were appointed for clinical accompaniment giving a ratio of one supervisor to 10 students.

In addition to the aim of widening access, particularly to disadvantaged students, the purpose of this foundation programme also essentially included providing supplementary foundation support in order to improve the throughput rate of students. It was unclear how these students experience the support of their lecturers offering the foundation programme offered at the Department of Nursing at a university in the Western Cape.

## Problem statement

The Bachelor Nursing Foundation Programme, since its commencement, had not undergone any formal reflective process with the aim of evaluating the supporting nature in the programme. Students in the foundation programme reflected in their year-end subject evaluation forms that they needed more support from their lecturers during their classes. The importance of engaging in reflection is described as giving deep thought to your teaching or learning; thinking about what you do in the classroom, why you do it and why it works; and enabling you to identify any changes and improvements you could make (Neill 2018). In 2015, a School of Nursing at a university of the Western Cape was formally unaware of how students experience the support from lecturers in the foundation programme. From the problem statement, the following question was posed:

What were the experiences of students of the support from lecturers in the foundation programme?

## Aim of the study

The aim of this study was to investigate the support that academics gave to students in the foundation programme at a university of the Western Cape.

## Research objective

The study aimed to explore and describe the experiences of students about the support from lecturers in the nursing foundation programme.

## Definition of key concepts

*Foundation or extended curriculum programme* is a programme that ‘provides for students to have an extended period of their first year studies over a period of two years’ (De Klerk et al. 2005:1). In this study, the foundation programme referred to a first year offered over 2 years in a 4-year degree nursing programme, thus an extended programme of 5 years.

*Academic* is a lecturer and nurse educator registered at the South African Nursing Council that offers courses over a period of 2 years in the foundation programme.

*Support* is an action in which nursing academics give assistance to students undertaking the foundation programme.

## Contribution to the field: Significance of the study

The findings of this descriptive study add insight to experiences of students in the foundation programme, to provide recommendations for lecturers to support students in a nursing foundation programme.

## Research design and method

A qualitative, exploratory, descriptive and contextual design was followed in this study as a means of exploring and understanding the experiences of individuals in the foundation programme ascribed to a social or human phenomenon (Creswell 2014), such as support in the foundation programme. An *exploratory research design* was followed as the researcher wanted to gain insights into the phenomenon of support of lecturers to foundation students of which little was known (Creswell 2014). The *descriptive design* was interested in the process and understanding that were gained by means of words (Creswell 2014). The researcher conducted the study of the experiences of students on the support of lecturers in terms of the immediate context and it involved far more than the physical environment. The method of individual interviews was followed to collect data.

### Population and sampling

As it was not possible to reach all the members of the target population, the researcher had to identify that portion of the population which was accessible, in terms of transportation and finance (Creswell 2018). The accessible population was 51 students who were registered in the second year of the extended foundation programme. *Purposive sampling* allowed for selecting participants who have experienced the nursing foundation programme. The eligibility criteria for this study were thus students who were currently registered for an extended 5-year programme and had completed the first year of the programme. From the accessible population, participants were interviewed at which stage data saturation was achieved with the eighth participant.

### Data collection

An information session was arranged with the students and dates of availability determined with the lecturer, not to interfere with the programme. *Semi-structured interviews* allowed the researcher to gather in-depth information about the students’ experiences (Van der Walt, Brink & Van Rensburg 2016) of the support of lecturers. The data-gathering instrument was an *interview guide* that was seen as the most direct method of obtaining facts from the participant (Van der Walt et al. 2016). One-on-one interviews allowed the researcher to observe the students’ facial expressions and body language in support of their verbal responses to the interview questions. *Observational data* were captured as field notes where observational data refers to raw materials an observer collects from interviews (Leedy & Ormrod 2014). All interviews were conducted at a time that was convenient for both the researcher and the student, in a private room without any interruptions. Interviews took around 30 min each. Informed written consent was obtained from the participants for the use of digital recording and for taking written notes.

### Data analysis

Open coding was followed, and the process created order, structure and meaning to the volume of data collected. All preconceived notions and ideas were avoided and eliminated by the researcher.

An interpretive approach was applied during the data analysis process. This approach implied that data were described and reported on what was seen, heard and understood. Data were transcribed, read, re-read and arranged in themes and categories by the researcher. An independent coder met with the researcher during a consensus meeting and finalised the analysis.

## Rigour of the study

Trustworthiness referred to the findings that were a true reflection of the personal or lived experience of the phenomenon under investigation (Van der Walt et al. 2016). For credibility, the contextual determinants were described in detail and field notes provided additional contextual information. The researcher made use of member checks to verify data that had been analysed adequately. The researcher went back while analysing the data to one student to ensure that she heard correctly on the recorder. The research process was documented, logical and traceable for purposes of dependability. An independent coder confirmed the coding with the researcher during a consensus meeting. For transferability, the researcher ensured that a thick description of the data was given and that all findings were well explained.

## Ethical considerations

Ethical clearance was obtained from the Senate Research Committee of the University of the Western Cape, reference number: 12/6/32. Access to the participants was negotiated by submitting the ethical clearance letter to the Head of the Department of Nursing of a university in the Western Cape for permission to conduct the study, and the aims and objectives of the study were explained to them. Information sheets were given to all participants, explaining the purpose of the study and what would be expected of them in order to participate. Written informed consent was obtained from the participants. All participants had a choice whether to participate in the study or not. They were informed of their rights to withdraw from the study at any time and were assured that the withdrawal would in no way affect their studies. There were no known risks in participating in this study; however, interviews were held in a private office and did take the time period into consideration. No risks were foreseen, although a psychologist was on the university premises during the time of the interviews if an emotional need arose to refer a student for counselling.

Participants were assured of the confidentiality of what they would share. In this study, confidentiality was ensured by the use of codes to represent the participants on transcripts and not their names. The researcher ensured that access to the data was restricted to the researcher, the independent coder and the supervisor. The identity and privacy of students were protected and respected during the course of the research.

## Results and discussion

### Demographic data

The target population included all male and female students registered for the B. Nursing 5-year Foundation programme at a university in the Western Cape. All participants completed the first year of the programme. The participants included two males and six females with ages ranging from 20 to 51 years (*n* = 8). Three of the participants were Coloured and five were black. Three participants’ home language was isiXhosa, two spoke seTswana as their mother tongue, one participant was Afrikaans and two English-speaking participants.

### Themes and categories

The findings indicated a theme on creating an overall supportive learning environment. The theme is presented in categories and subcategories (Table 1).

**TABLE 1:** Theme and categories.

Theme	Category	Sub-category
Creating an overall supportive learning environment	The supportive role of the lecturers	Open and approachable attitude Caring and nurturing Advisors Persistent and patient during support
	Promoting peer support	Support extended beyond the programme Provided a sense of camaraderie

### Theme: Creating an overall supportive learning environment

#### Sub-theme: The supportive role of lecturers

Students experienced a generally supportive environment during the programme. They experienced lecturer support as being beneficial and contributing to their academic success. Personal and professional characteristics of lecturers played a vital role in the support offered to students. According to Guyana Ministry of Education (2017), the best teachers care about their students and are passionate about the material they teach. Passion and enthusiasm draw students into learning as it excites their minds, creates curiosity and inspires them to raise their energy levels in class (Guyana Ministry of Education 2017).

**Open and approachable attitude:** A teacher should possess awareness and responsiveness to students’ needs and should interact with students as individuals and in class (Dobrovská & Andres 2016:42).

Students felt comfortable with lecturers as they could ask the lecturer anything they were unclear about and thus felt supportive:

‘I always felt at ease and like I could ask the lecturer for anything.’ (P7, 22 years old, female)

‘I felt at ease with the lecturer and I felt like I could approach the lecturer about anything.’ (P6, 20 years old, female)

A good teacher is someone who is approachable, engaging and inspiring, and who has a sound knowledge of theory of what they are trying to teach (Kumari et al. 2016:5436). Students can see lecturers as approachable when they support students in their study progress by being available for students who seek assistance and when students feel that teachers can be trusted and are willing to listen to their problems (Hagenauer & Volet 2014:378).

Students expressed that they felt supported through a trusting relationship built with by the lecturers, and thus felt comfortable to disclose private information of their past to the lecturers, seeking support:

‘… you did not feel scared talking about your past … you actually went deep into your past.’ (P2, 51 years old, female)

The Guyana Ministry of Education (2017) found that teachers who respect their students’ privacy and who are sensitive to their students’ needs and feelings tend to be the most respected.

Students also described their lecturers as supportive and always willing to assist, making time and sharing their knowledge with them:

‘… they were very helpful and supportive … they were like open. We could come to the offices and then they would share their knowledge and experience.’ (P3, 23 years old, female)

‘… always available when we needed to ask something, and he also gave a little bit extra, you know, like when he explained stuff, and with feedback on assignments and tests.’ (P5, 25 years old, male)

Komarraju, Musulkin and Bhattacharya (2010:339) note that students also value the time that lecturers may spend with them outside the classroom, as well as any career development advice they may provide.

**Caring and nurturing characteristics:** Caring can be viewed as fostering a sense of belonging, getting to know students personally, supporting academic success and attending to physiological needs (Garza et al. 2014:1).

Students experienced the support of lecturers in a nurturing and caring manner, and appreciated the individual time lecturers spend with students needing extra assistance:

‘… the lecturers were very supportive … that was the … the … one of the support that we got. They will tell you … they introduced us to the problem. … They will sort of nurture you. …’ (P1, 21 years old, female)

‘He really cared about each student, and spent individual time with us when we needed help.’ (P6, 20 years old, female)

The caring teacher is someone who holds relationships at the centre of their encounters with students. It is the teachers’ beliefs that students are being empowered and being made intellectually richer, because of the particular types of interactions they have with them (Walker & Gleaves 2016:65–66).

**Advisors:** Advising and guiding students are core responsibilities of lecturers. Students have different abilities and capabilities that teachers need to advise on (Botty et al. 2015:113).

Students experienced the lecturers as advisors who assisted them with problems and concepts that were unfamiliar and, in a sense, guided them through their studies:

‘ … having to solve problem, understand concept that you were unfamiliar to, sort of groom you … advising you.’ (P1, 21 years old, female)

Lecturers must possess positive supportive characteristics so that students can easily approach them with confidence if they have difficulty in solving tasks or if they have not fully understood the concepts taught (Botty et al. 2015:113).

**Persistent and patient during support:** Kumari et al. (2016:5436) describe the highly effective teacher as someone who supports the student by both understanding the content and explaining it in the students’ language.

Students found that lecturers were always persistent and patient when assisting them with difficult concepts, and they added detail where appropriate so that the students could better understand the concepts.

‘… they will go into detail when you don’t understand a concept … and teach you until you get the concept.’ (P1, 21 years old, female)

A teacher has to be patient, methodical and smart enough to reach out to students on all learning levels and communicate concepts with confidence and clarity so that all students will be able to understand (Kumari et al. 2016:5436).

Lecturers were willing to go the extra mile and spend extra time with students, to ensure that every student understands the work:

‘… they were brilliant, in the sense that … uhm … they were always willing to go the extra mile (uhm) especially when there was certain uncertainties ….’ (P2, 51 years old, female)

‘They did support us if we struggled … (uhm) … and they had a lot of time for us … they will take you step by step if you didn’t understand, like really have a lot of patience with you … explain it over and over ….’ (P4, 22 years old, male)

‘… spent extra time with us, like to explain things and so on, and made sure that we understand things… He was patient and he listened when we had questions, and he always had time for our problems.’ (P7, 22 years old, female)

A study indicated that an empathetic lecturer can motivate unenthusiastic students to discover their maximum potential through consistent encouragement and self-assurance (Hiew 2012:16).

#### Sub-theme: Promoting peer support

Peer support was found to be a vital component of teaching methods to successfully completing the programme. As the foundation year consisted of 51 students, they were a small, intimate group, who shared a lot of similarities and experiences and were thus closely knit. Students felt that peer support was encouraged by lecturers and assisted them to succeed in their studies. Without this teaching method, they might have not come so far:

‘… anyway with persistence … they were actually helping me to push through to ….’ (P2, 51 years old, female)

‘… like my colleague that was studying with … We were able to help each other with. … (uhm) … problems … my friend and I would sit and work and solve problems and do the work that we were told to do. We practice and commitment so we manage to flow through the foundation year. … We were a very intimate group. Support system was very good, we have done things quicker.’ (P1, 21 years old, female)

According to Zheng and Li (2016:76), particularly in the case of adult students, the lecturer should focus on encouraging students to learn from their peers and can in this way best capitalise their experiences and knowledge.

Lecturers also focused on classroom engagement to enhance learning. The older students appreciated the support from the group, as their fellow students were open and approachable and made time to listen to them and assist them where needed. For some students, the group felt more like a family, as they were closely connected to each other, and they could share intimate details:

‘… but I latched on with the younger ones you know and you think for yourself, this is for your own gain, this child getting 80/90% in the class, you go just to say ‘Hi what is your name?’ and you know … and hug them and everyone addresses me as ‘aunty’. So you know Aunty Joey, no man … I said come and show me now this … they said no, not like that … they actually made time for me … and I could say that actually applauded them ….’ (P2, 51 years old, female)

‘… we were very connected… like a family … We were a small group, but we are connected to one another. We are able to shared things … and I was able to connect to them.’ (P3, 23 years old, female)

According to Kiefer, Alley and Ellerbrock (2015:2), peer emotional support helps to promote students’ sense of belonging and may provide a foundation for academic motivation and classroom engagement.

**Provided a sense of camaraderie:** In the foundation year, students found that they were enabled to build lasting relationships and friendships with each other because they could identify with each other and they had a sense of belonging.

They shared a history, especially those students who were in their final year:

‘… so we started building you know a good relationship with each other … that is still lasting … we identified a lot with each other even now in our final year and just … you know … remind each other … remember where we come from, how we revel, how we … you know, that “kamaradeskap”, and everyone was looking out for each other.’ (P2, 51 years old, female)

According to Won, Wolters and Mueller (2017:1), the sense of belonging to school can be related to teaching of mastery goals, whereas a sense of belonging to peer groups relates with learning to set performance goals by lecturers.

## Limitations of the study

The study was qualitative in nature, and the findings cannot be generalised to the broader population in other settings.

## Recommendations

Students appreciate the support of lecturers in different ways. Lecturers should focus on being open and approachable to individual counselling or group discussions that can identify and reduce potential worries and stresses that the students may have. Building self-confidence of students during the foundation programme in a bachelor’s degree could be enhanced by, for example, lecturers praising students for work well done. Praise could be used to motivate someone to perform at a continuously higher level of learning. In this way, it could be applied by lecturers to strengthen an individual’s self-image. Through the years, lecturers have traditionally praised students when their work is considered to be praiseworthy, and the rationale for this is that the students will then be motivated to continue in the same positive direction.

A caring and nurturing environment should be essential. Lecturers should have an informative session on how the foundation year fits into the overall programme. As first-year nursing students, their sense of belonging can be enhanced by receiving the help and guidance needed to find their way in the new environment.

Academics are advisors and should assist students by providing computer literacy and basic sciences classes, and encouraging students to attend all tutorials and the campus orientation, as it will be of great benefit to them. According to Nathan (2015:162), academic support is vital in higher education, and tutorials are a form of such academic support. Although adult-centred learning can be considered as self-directed, guidance is required to meet the academic achievement of higher education students. Academics should have patience and are in a position to stay persistent and provide opportunities to encourage student development, and they need to consider the best means to ensure efficient and effective student support. Peer support is crucial and lecturers could incorporate tutorials in all subjects for group work, as this could form the basis of academic support. This process can also be enhanced by having smaller group activities within the class, to practise participative teaching and make use of student presentations. Every foundation-year student could be appointed to a first-year student mentor, thus forming a buddy system which will be of benefit to both students, in which the lecturer will promote an environment of camaraderie.

Future research can be conducted to re-evaluate the programme as a whole and measure how effective it is in preparing students for the mainstream programme, possibly by looking at throughput and attrition rates of foundation programme students, and perhaps comparing these data with students that entered the mainstream nursing programme. Research can be conducted on this topic at other universities in South Africa offering the foundation programme, which then could be compared with the findings of this research.

## Conclusion

The findings and discussions brought to the fore that the foundation programme offers a generally supportive learning environment, in which lecturers and colleagues or peers play an important supportive role.

The results of this study indicate that the support of the lecturer could result in multiple levels of growth, both personally and academically – this was more among the students who were generally older and more mature. Students viewed the lecturer in the foundation year as helpful that created an environment of positive teaching and learning. Overall, the experiences were viewed as having a generally supportive environment of lecturers and students interaction.

## Footnotes

1 The author understands and acknowledges the sensitivity of the terms in use and prefers to use as classified according to STATS SA and the South African government in order to avoid misinterpretation of the study results.
